# Comparative Analysis of the Offensive Effectiveness in Winner and Losing Handball Teams

**DOI:** 10.3389/fpsyg.2020.547110

**Published:** 2020-09-25

**Authors:** Willian Ferrari, Gonçalo Dias, Tiago Sousa, Hugo Sarmento, Vasco Vaz

**Affiliations:** ^1^Faculty of Sport Sciences and Physical Education, University of Coimbra, Coimbra, Portugal; ^2^Research Unit for Sport and Physical Activity, Faculty of Sport Sciences and Physical Education, University of Coimbra, Coimbra, Portugal

**Keywords:** match analysis, performance analysis, observational methodology, team sports, handball analysis

## Abstract

The purpose of this study was to determine differences related to the offensive process between winning and losing teams among teams participating in the European Handball Federation Champions League (EHFCL) in 55 matches across five seasons. The key indicators used in this study are the offensive actions, team possession type and the zones of the field, goals, and shooting effectiveness. A total of 34 indicators were analyzed and compared using Mann–Whitney U tests. Sixteen key indicators are identified to confirm differences both from the aspect of the collective game in terms of assists (9.10 ± 2.75 vs. 7.29 ± 2.65), goals of positional attack (21.38 ± 4.60 vs. 18.20 ± 3.62) and from the aspect of individual goals from 6 m (16.67 ± 3.98 vs. 13.64 ± 3.70), and the effectiveness of shots (68.19 ± 6.83 vs. 59.41 ± 6.33). Winning teams performed better regarding the variables that defined the effectiveness of offensive shots, especially successful positioned attacks and fast attacks. They also had a greater number of assists. The profiles of the most successful teams can help coaches and practitioners to achieve better performances adjusting the training process according the performance indicators that seem to lead more often to success.

## Introduction

Handball is a complex sport in which players’ performance can be analyzed and presented in various manners (Skarbalius et al., 2013). Additionally, is considered a transition game, as players often alternate between defensive and offensive play and the actions of the match are characterized by alternations between running and sprinting (Curiţianu et al., 2015). Technical skills, anthropometric characteristics, and high levels of muscle strength and speed are the most important factors in gaining an advantage in elite handball competitions (Rannou et al., 2001; Gorostiaga et al., 2005). However, success in collective sports requires that a team integrate many factors beyond physical factors (Smith, 2003).

Among these other success factors is match analysis (Hughes and Bartlett, 2002). Over the years, match analysis has evolved very significantly in several sports, such as football (Sarmento et al., 2014b, 2018a), futsal (Agras et al., 2016), or basketball (Courel-Ibáñez et al., 2017). Nerveless, the investigation in handball match analysis is not well established in the researcher’s scientific agenda (Ferrari et al., 2019).

More specifically, past research in this scientific area reveal the interest of the scientific community in detecting the differences between winners and losers’ teams. Comparisons between offensive and defensive actions, goals or points, interactions between players, and assists are the most commonly used factors assessed by researchers in basketball (Blake, 2015; Madarame, 2017, 2018), football (Lago-Peñas, 2007; Szwarc, 2007; Rumpf et al., 2017; Sarmento et al., 2018b), female handball (Ohnjec et al., 2008; Costa, 2018), and other collective sports (Vaz et al., 2011; García-Marín and Argudo Iturriaga, 2017).

Specifically, in male handball, Skarbalius et al. (2013) concluded that goals, effectiveness of positional attacks, and shooting efficiency in the offensive process were important performance indicators that distinguished winner and losers teams. On the other hand, Vuleta et al. (2015) concluded that there are six offensive indicators that differentiated winners from losers: goals at 6 m, total goals, total shots, shots at 9 m, counterattacks, and assists. Additionally, Ferrari et al. (2014) also concluded that positional attack, penalties of 7 m, and 9-m shots and their effectiveness were associated with winner teams. Also, Meletakos et al. (2011) showed that the 6- and 9-m throws strongly impacted the offensive profile of the teams. In particular, 6-m efficacy remained constant in all the competition analyzed in their study, while 9-m effectiveness increased significantly over the years.

Contrary to what happens in other sports, as in football (Sarmento et al., 2014a, 2018a), futsal (Agras et al., 2016), or basketball (Courel-Ibáñez et al., 2017), the available literature on match analysis in handball is still scarce and focused mainly in four variables of performance: total number of shots and their effectiveness, match outcome, Time Outs, and the analysis centered in home advantage (Ferrari et al., 2019). In this sense, and according to (Meletakos et al., 2011; Prieto et al., 2015; Román, 2015), the existing handball performance analysis database is insufficient to allow coaches and match analysts to establish performance optimization criteria, and additional investigation is needed in order to better understand the specific influence of different performance indicators in the performance of the teams.

In this sense, the aim of the present study was to determine the differences between winning and losing teams participating in the European Handball Federation Champions League (EHFCL) of men’s handball in terms of their offensive processes (offensive type of possession, shoots, goals scored, interactions, assistances, turnovers, and punishments) using notational analysis from five sporting seasons.

## Materials and Methods

### Sample

We used data from the teams that participated in the EHFCL in the competitive moments of the quarterfinals and Final Four (*n* = 55) over five seasons (2012/2013 to 2016/2017). Only matches in which there was a winner were included. The teams that compete in this competition are considered the best club teams in the world. The selection to participate in this competition stems from the fact that they have won the respective national championships, representing the EHFCL as the main competition at European level of clubs, being also considered the most difficult competition at the level of clubs worldwide. Naturally, these teams are made up of a large majority of those who are considered the elite athletes worldwide.

### Measures

**FIGURE 1:**
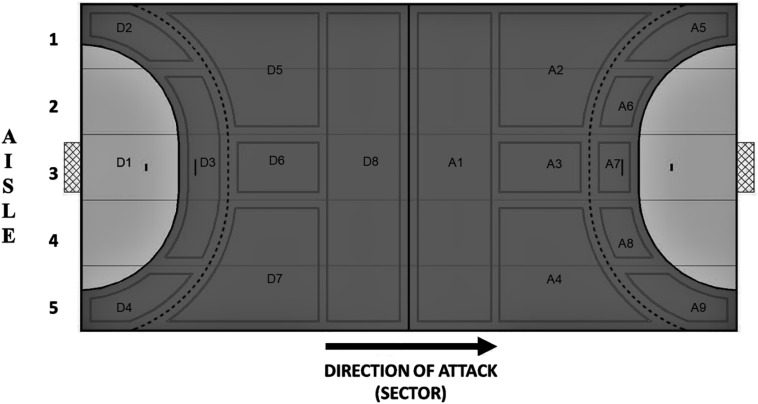
Field zones divided into 17 zones and five aisles with the numbering of zones designated according to the direction of the attack. D, defense; A, attack.

The observational instrument tool used to collect data is developed and validated by Ferrari et al. (2018b) and included a combination of offensive actions (organized attacks, fast attacks, counterattacks, total shots, and total goals), team possession types (Table 1) and field of action (i.e., field zones; Figure 1). Data collection and analyses were conducted according to the Declaration of Helsinki.

**TABLE 1:** Description of variables and definitions of categories used in the team match performance analysis.

Variables and Categories
**Team Possession Type**
Positional attack – An action is considered when each player occupies their specific position and initiates interactions to move the defense, this phase begins when the opponent’s defense is established in their position, against an organized offensive system.
Fast attack – This is considered as a second offensive chance, made by later players in the defensive system, who progressed in the field with speed, through quick passes to the attack, in order to create a situation of superiority or defensive disorganization of the attack to opponents’ team.
Counterattack – This offensive method starts in the defensive field, trying to get as fast as possible to the opponents’ goal with as few passes as possible.
**Type of Offensive Actions**
Collective actions Type-I – Complete collective actions (e.g., start, progression, and completion) are those that result from dynamic or static play, implying a start, a progression development in the field of play for more offensive areas and a finalization of the offensive sequence (with or without efficiency).
Collective actions Type-II – Incomplete sequence, that result from loss of ball due to technical or tactical error.
Collective actions Type-III – Actions that start by a stopped ball situation (e.g., 7 m penalty shot, direct or indirect free kick, foul, etc.) that imply a short finalization and imply a rapid finalization of the offensive process (less than three passes between the players).
**Finishing Zones**
Before 9 m – Any action that was completed before the dashed line of the 9 m represented in Figure 1 (A1, A2, A3, and A4).
Between 9 and 6 m – Any offensive action that was completed after the 9-m dashed line represented in Figure 1 (A5, A6, A7, A8, and A9).
Defense zone – Any action of the offensive process that has been completed in the zone of defense represented in the figure as all zones containing “D.”
**Shooting Zones**
9 m – The player making the shot has his last support foot placed before the dashed line.
9–6 m – The player who hit the ball had his support foot touching the ground, between the dashed line (9 m) and the 6 m.
6 m – The player, with his jump, invades the airspace of the area, where he had to finish before landing.
7 m – It was carried out while 7 m penalty shot was awarded.
Defense – When the shot was taken from the field of defense of the team.
**Effectiveness**
With efficiency – Shot with a goal scored.
Without efficiency – Recovery of ball possession by the opponent, ball out, violation of the rules of the game, shot defended by the goalkeeper, shot out, shot into the opponent.

In addition to the means of observation mentioned above, effectiveness will be analyzed in two ways: (1) total efficacy, which is the ratio of total actions to their respective variables, and; (2) finishing efficiency, which is the ratio of the total number of goals scored relative to the total number of shots taken.

Data was collected for winning and the losing teams of matches simultaneously using Video Observer ^®^ software. Then, the data were entered into Microsoft Excel 2013 for further analysis. Each sequence has been analyzed in sequential way.

To ensure the reliability of the observations, intra- and inter-observer agreement were used for all the criteria, as stipulated by the Cohen’s Kappa index (Cohen, 1960; Saavedra et al., 2018), which was greater than 0.91. For that, two experienced handball analysts in match analysis procedures used the specific observational instrument tool to analyze the selected offensive sequences. After a training period, each analyst had analyzed around 10% of the offensive sequences randomly selected, in order to analyze the interobserver reliability. Intraobserver reliability was completed using the offensive sequences of the same offensive sequences, but the lead author of this study repeated these on two occasions (after a 4-week period).

### Statistical Analysis

The characterisation of the sample was produced through descriptive statistics using the parameters of average central tendency and dispersion (standard deviation and amplitude) to extract information regarding the general dynamics of handball matches (Bajgoriæ et al., 2017).

Non-parametric statistical analyses were performed using Mann–Whitney U tests, which identified a subset of variables related to the game that distinguishes the teams that have won from those that lost in each of the five EHFCL seasons. Cohen’s d effect size was calculated and considered small (*d* < 0.2), moderate (0.2 < *d* < 0.6), big (0.6 < *d* < 1.2), very big (1.2 < *d* < 2.0), or nearly perfect (2.0 < *d* < 4.0; Cohen, 1988). The level of statistical significance was set at 0.05, and all analyses were performed in IBM SPSS Statistics (24.0).

## Results

The results were distributed according four aspects of the offensive process: (1) the teams’ offensive actions, (2) the goals scored by the teams, (3) goals scored in different finishing zones, and (4) the effectiveness of actions when a goal was scored.

Regarding offensive actions presented in Table 2, we concluded that winner teams scored more goals through positional attacks, and trough Action Type I, scored more goals (in total and from 6 m line), and performed more assistances.

**TABLE 2:** Results of the Mann–Whitney U test for offensive actions.

	Winners (*n* = 55)	Losers (*n* = 55)	Different from the Winning Team	Z	*p*	*d*
	
	Mean ± SD (Min–Max)					
Actions	53.44 ± 6.01 (45–76)	53.40 ± 5.99 (45–76)	0.04	−0.51	0.95	0.00
Positional attack	40.52 ± 5.37 (30–45)	40.47 ± 5.02 (32–55)	0.05	−0.75	0.94	0.01
G. Positional attack	21.38 ± 4.60 (12–31)	18.20 ± 3.62 (12–30)	**3.18**	–**3.86**	**0.00**	**0.76**
Counterattack	5.52 ± 2.77 (1–16)	4.81 ± 2.41 (1–11)	0.71	−1.29	0.20	0.27
G. Counterattack	4.24 ± 2.38 (1–13)	3.45 ± 1.68 (0–8)	0.79	−1.64	0.10	0.38
Fast attack	7.38 ± 4.03 (1.17)	8.10 ± 4.33 (0–18)	−0.72	−0.99	0.32	−0.17
G. Fast attack	4.35 ± 2.39 (0–11)	4.27 ± 2.66 (0–11)	0.08	−0.47	0.64	0.03
Type action – I	35.38 ± 5.69 (24–56)	34.72 ± 6.41 (22–55)	0.66	−0.85	0.39	0.11
G. Type action – I	24.53 ± 4.38 (15–35)	21.09 ± 4.20 (13–34)	**3.44**	**−4.10**	**0.00**	**0.80**
Type action – II	9.80 ± 3.42 (4–20)	10.05 ± 3.30 (4–20)	0.12	−0.47	0.64	−0.07
Type action – III	8.16 ± 3.26 (2–16)	8.61 ± 2.80 (2–17)	−0.25	−0.90	0.37	−0.15
G. Type action – III	5.44 ± 2.70 (1–12)	4.84 ± 2.58 (1–12)	0.60	−1.15	0.25	0.23
Shoots	45.47 ± 5.78 (33–66)	45.16 ± 6.62 (33–70)	0.31	−0.07	0.94	0.05
Total goals	29.96 ± 4.40 (22–43)	25.93 ± 4.24 (15–39)	**4.03**	–**4.80**	**0.00**	**0.93**
Interactions	654.73 ± 84.72 (461–845)	661.09 ± 84.36 (458–818)	−7	−0.31	0.76	−0.08
Assistance	9.10 ± 2.75 (3–16)	7.29 ± 2.65 (2–14)	**1.81**	–**3.33**	**0.00**	**0.67**
Turnovers	9.80 ± 3.42 (4–20)	10.05 ± 3.30 (4–20)	−0.25	−0.47	0.64	−0.07
Punishments	3.76 ± 1.62 (0–8)	3.96 ± 1.80 (0–9)	−0.20	−0.62	0.53	−0.12
G. 9 m	5 ± 2.76 (1–12)	4.96 ± 2.72 (1–12)	0.04	−0.60	0.95	0.01
G. 9–6 m	5.05 ± 2.31 (0–11)	4.49 ± 2.04 (1–10)	0.56	−1.36	0.17	0.26
G. 6 m	16.67 ± 3.98 (10–29)	13.64 ± 3.70 (7–25)	**3.03**	–**3.46**	**0.00**	**0.79**
G. 7 m	2.98 ± 1.80 (0–7)	3.07 ± 1.76 (0–8)	−0.09	−0.39	0.69	−0.05
G. Defense zone	0.24 ± 0.51 (0–2)	0.20 ± 0.45 (0–2)	0.04	−0.28	0.78	0.08

*G, Goals. Bold values are represented as statistical significance.*

Table 3 presents the results concerning the comparison relationships between goals in the different offensive areas of the field zone. It is important to highlight that winner teams scored more goals from the central (A7) and right (A8 and A9) zones of the offensive midfielder when compared with the loser teams. For both winner and loser teams, the central zones of the offensive midfield (A6, A7, and A8) were the zones from where the most goals were scored.

**TABLE 3:** Results of the Mann–Whitney U test for goals scored in the final zones of action.

	Winners (*n* = 55)	Losers (*n* = 55)	Different from the Winning Team	Z	*p*	*d*
	
	Mean ± SD (Min–Max)					
A1	00 ± 00 (0–0)	0.09 ± 0.44 (0–3)	0.09	−1.75	0.08	−0.29
A2	1.15 ± 1.21 (0–4)	1.05 ± 1.11 (0–5)	0.10	−0.27	0.79	0.09
A3	2.55 ± 1.96 (0–7)	2.60 ± 2.01 (0–8)	−0.05	−0.15	0.88	−0.03
A4	1.29 ± 1.21 (0–6)	1.24 ± 1.22 (0–5)	0.05	−0.34	0.74	0.04
A5	2.35 ± 1.65 (0–7)	2.11 ± 1.94 (0–12)	0.24	−1.06	0.28	0.13
A6	5.64 ± 2.39 (1–11)	4.78 ± 2.03 (1–11)	0.86	−1.82	0.06	0.39
A7	9.11 ± 2.84 (2–14)	7.75 ± 2.89 (2–15)	**1.36**	−**2.66**	**0.00**	**0.47**
A8	5.42 ± 2.34 (1–10)	4.18 ± 2.40 (0–12)	**1.24**	−**2.82**	**0.00**	**0.52**
A9	2.24 ± 1.37 (0–6)	1.73 ± 1.35 (0–6)	**0.51**	−**1.92**	**0.05**	**0.37**

*A, attack zones. Bold values are represented as statistical significance.*

Regarding the effectiveness of the winning teams’ victories (Table 4), the total number of shots performed in the match was compared to the number of goals scored. Winner teams seem to be more effective than loser teams in all the zones between the 6- and 9-m lines. Additionally, the results showed that winner teams were more effective in scoring goals through positional and fast attacks, and trough type actions I and II. It is interesting to note that the effectiveness for goals scored trough counterattack situations and from 7 m zone were similar between winner and loser teams.

**TABLE 4:** Results of the Mann–Whitney U test for the effectiveness of shooting.

	Winners (*n* = 55)	Losers (*n* = 55)	Different from the Winning Team	Z	*p*	*D*
	
	Mean (%) ± SD (Min–Max)					
Total Effectiveness	68.19 ± 6.83 (54.55–85.37)	59.41 ± 6.33 (44.11–75.60)	**8.78**	**−6.32**	**0.00**	**1.33**
9 m	46.80 ± 13.12 (16.67–71.43)	39.64 ± 15.87 (11.11–83.33)	**7.16**	**−2.86**	**0.00**	**0.49**
9–6 m	55.35 ± 17.11 (0–83.33)	47.99 ± 15.44 (12.50–80)	**7.36**	**−2.53**	**0.01**	**0.45**
6 m	75.53 ± 8.95 (53.57–95)	70.43 ± 10.89 (52.63–94.44)	**5.10**	**−2.78**	**0.00**	**0.51**
7 m	79.95 ± 21.94 (0–100)	74.94 ± 23.92 (0–100)	5.01	**−**1.32	0.23	0.22
Defense Zone	78.20 ± 38.12 (0–100)	58.89 ± 47.50 (0–100)	19.31	**−**1.06	0.29	0.45
Positional attack	65.55 ± 9.03 (44.45–82.86)	56.32 ± 8.17 (41.17–75)	**9.23**	**−4.97**	**0.00**	**1.07**
Counterattack	84.92 ± 16.86 (33.34–100)	83.94 ± 19.14 (33.33–100)	0.98	**−**0.16	0.99	0.05
Fast attack	74.75 ± 22.50 (0–100)	67.22 ± 19.78 (16.67–100)	**7.53**	**−2.53**	**0.01**	**0.36**
Type action – I	69.67 ± 7.26 (55.55–89.28)	61.13 ± 7.13 (46.43–77.28)	**8.54**	**−5.40**	**0.00**	**1.19**
Type action – III	65.28 ± 17.83 (16.67–100)	54.23 ± 19.87 (16.67–100)	**11.05**	**−3.30**	**0.00**	**0.59**

*Bold values are represented as statistical significance.*

## Discussion

The aim of the present study was to analyze the differences in the offensive process between winning and losing teams participating in the EHFCL of men’s handball. Results showed that the most common offensive method used by the analyzed teams is the positional attack. Nerveless, when in situations of numerical superiority, there exist a tendency to develop more offensive sequences trough situations of counter-attacks and fast attacks (Prieto, 2015).

The only game-action-related variable for which there is a significant difference between winners and losers is the number of assists (which are considered as the final pass before a definite chance at a goal). This performance indicator has been associated in previous studies (Gutiérrez Aguilar and López, 2010; Vuleta et al., 2015), with the winning teams. However, the same authors concluded that counterattacks are related to winning, which was not found in the present study. Possibly, the very high level of the sample of this study, which includes teams that are finalists in a European championship, may contribute to the absence of such a marked difference in counterattack situations.

Goals scored differentiated winners and losers, with specific key indicators being goals scored at 6 m, situation of match in which there is always a more significant advantage for the attacker (Hatzimanouil et al., 2017). Additionally, winning teams scored more goals during attacks that started with the opponents’ defense already fully organized, probably due their superior capacities to resolve offensive problems even against teams well organized in their defense (Rogulj, 2000; Ferrari et al., 2014).

Regarding the goals scored trough Collective Action Type-I, the results showed that winning teams are more balanced than losing teams in all aspects of the offensive. These results are in line with those reported by Ferrari et al. (2018a).

Concerning the finishing zones, the winning teams present higher levels of effectiveness from 6 m line, when compared with the losing teams. Additionally, they scored more goals than losing teams from zones A7 (central zone), A8 (lateral area), and A9 (wing zone), which are all close to the 6-m line on the attacking team’s right-hand side. These finding conflicts with the results of Prudente (2006), who found that winning teams scored more goals from the left-hand side.

Shooting effectiveness at 9 m and at 6 m, exhibited the largest differences between winners and losers. Vuleta et al. (2015) analyzed indicators of team efficiency and also identified shots at 6 m as a distinguishing factor between winners and losers. Moreover, Meletakos et al. (2011), who evaluated performance indicators at the world championship level, showed a total shot effectiveness of 55% in favor of winning teams, which is well below the levels presented in this study. In this sense, the results of our study seem to show that the analyzed competition presents higher values for the total effectiveness of shots performed by both teams (winners and losers), which is probably due to the high level of the teams observed.

Within the organization of a team, the results showed that winning teams outperformed losing teams in terms of positional attacks and fast attacks. This was also true of effectiveness in Action Collective Type-I and Action Collective Type-III, which are finishing actions originating from a foul at 9 m and following a shot or a penalty shot from 7 m, respectively.

A possible limitation of this study is the fact that the difference in goals at the end of each match was not considered. The introduction of this fact may have been taken into account in future studies in order to identify the existence of performance factors associated with balanced vs. unbalanced matches as suggested by Lupo and Tessitore (2016).

## Conclusion

The objective of this investigation was to determine the main indicators that distinguish winning teams from losing teams in European men’s club handball using data from games played over the last five years. Differences were found between several performance indicators. Nerveless, the effectiveness of shots was one of the profound differentiators of winning and losing teams.

To the best of our knowledge, this is one of the few studies that includes a multidimensional approach to some performance indicators that have rarely been studied in the past (e.g., shots performed between 6 and 9 m zone, analysis by type of possession, type of collective actions, etc.) in this sport. In this sense, this study can help coaches and practitioners to extracting more detailed data from the game that may be useful for adapting their training/game processes.

Thus, this study determined that victories are typically achieved by teams that performed better in different aspects of the offensive process and their effectiveness. In this sense, positional attack seems to be the most effective type of play. Therefore, coaches should seek to train situations that create more options and variants in their positional attack to make them increasingly effective. The training of situations of counterattack and fast attack should not be neglected either, given their importance in certain circumstances of the game.

This study presents a systematic analysis of matches that could be used to adapt the training process in order to improve the performance of teams/players. The profiles of winning teams can help coaches to achieve better sports success by focusing on the indicators detailed in this survey when training their teams. Additionally, this type of systematic analysis, prove to be useful in order to help coaches (and technical staff) to analyze their own/opponents teams in order to detected weaknesses/strengths and to adapt specific strategies accordingly.

## Data Availability Statement

The raw data supporting the conclusions of this article will be made available by the authors, without undue reservation, to any qualified researcher.

## Author Contributions

WF, VV, TS, HS, and GD conceived and designed the study and contributed to drafting the article and/or its critical revision and final approval of the version to be published. WF collected the data. WF, TS, and HS analyzed and interpreted the data. All authors of the submitted manuscript certified that they have sufficiently participated in the work to take responsibility for the manuscript’s content. Furthermore, each author certified that this material has not been and will not be submitted to or published in any other publication.

## Conflict of Interest

The authors declare that the research was conducted in the absence of any commercial or financial relationships that could be construed as a potential conflict of interest.
